# Is Sjögren’s syndrome dry eye similar to dry eye caused by other etiologies? Discriminating different diseases by dry eye tests

**DOI:** 10.1371/journal.pone.0208420

**Published:** 2018-12-03

**Authors:** Denny Marcos Garcia, Fabiola Reis de Oliveira, Carolina Maria Módulo, Jacqueline Faustino, Amanda Pires Barbosa, Monica Alves, Eduardo Melani Rocha

**Affiliations:** 1 Department of Ophthalmology, Otorhinolaryngology and Head & Neck Surgery, Ribeirão Preto Medical School, University of São Paulo, Ribeirão Preto, São Paulo, Brazil; 2 Craniofacial Research Support Center, University of São Paulo, Ribeirão Preto, São Paulo, Brazil; 3 Division of Rheumatology, Department of Clinical Medicine, Ribeirão Preto Medical School, University of São Paulo, Ribeirão Preto, São Paulo, Brazil; 4 Department of Ophthalmology and Otorhinolaryngology, Faculty of Medical Sciences, University of Campinas, Campinas, São Paulo, Brazil; University of Pretoria, SOUTH AFRICA

## Abstract

**Purpose:**

Dry Eye Disease (DED) is part of several conditions, including Sjögren’s syndrome (SS) and no single test to diagnosis DED. The present study intends to evaluate whether a set of signs and symptoms of DED can distinguish: a) SS from other non-overlapping systemic diseases related to DED; b) primary and secondary SS.

**Methods:**

182 consecutive patients with DED were evaluated under five groups: SS, graft-versus-host disease (GVHD), Graves' orbitopathy (GO), diabetes mellitus (DM), glaucoma under treatment with benzalkonium chloride medications (BAK). Twenty-four healthy subjects were included as control group (CG). The evaluation consisted of Ocular Surface Disease Index (OSDI), Schirmer test (ST), corneal fluorescein staining (CFS) and tear film break up time (TFBUT). Indeed, a subset of DED patients (n = 130), classified as SS1, SS2 and nonSS (NSS) by the American-European Criteria were compared. Quadratic discriminant analysis (QDA) classified the individuals based on variables collected. The area under Receiver Operating Characteristics (ROC) curve evaluated the classification performance in both comparisons.

**Results:**

Comparing SS with other diseases, QDA showed that the most important variable for classification was OSDI, followed by TFBUT and CFS. Combined, these variables were able to correctly classify 62.6% of subjects in their actual group. At the discretion of the area under the ROC curve, the group with better classification was the control (97.2%), followed by DM (95.5%) and SS (92.5%). DED tests were different among the NSS, SS1 and SS2 groups. The analysis revealed that the combined tests correctly classified 54.6% of the patients in their groups. The area under the ROC curve better classified NSS (79.5%), followed by SS2 (74.4%) and SS1 (69.4%).

**Conclusions:**

Diseases that causes DED, and also SS1, SS2 and NSS are distinguishable conditions, however a single ocular tools was not able to detect the differences among the respective groups.

## Introduction

Dry eye disease (DED) is frequent ocular condition and occurs in association with several ocular and systemic diseases. Until recently, the term dry eye was not very popular or part of the medical literature [1]. The terms *keratoconjunctivitis sicca*, keratomalacia, keratitis, *sicca* syndrome, and even Sjögren syndrome were used as synonimum, until recently years, when efforts were made to clarify the subjacent causes, mechanisms and propose criteria to separate the diseases [2–6] A broad definition of DED is widely accepted and has being used since 2007 [7], and was revised by a group of international experts, recently [8]. However, it is unknown whether the diseases that cause DED are distinguishable from SS in terms of symptoms and signs. Moreover, it is unclear which tests are necessary to diagnosis DED in different diseases.

Among common chronic incurable conditions related to DED are the following: Sjögren’s syndrome (SS), Diabetes Mellitus (DM), Graves’ Orbitopathy (GO), Graft versus Host Disease (GVHD and chronic use of benzalkonium chloride (BAK) [4,5,9–12]. Although DM, SS and GO involve different hormones alterations (i.e.; insulin, sex hormones, and tiroxine), systemic changes, including metabolic and inflammatory profile are different, and the demography are also not similar [13,14]. In the same way, GVHD, previously called Sjögren-like syndrome demonstrated to be different in the demographic and clinical aspect from SS [9]. Iatrogenic dry eye associated to BAK chronic exposure has been recently recognized and is related to corneal denervation, ocular surface inflammation and corneal. [12,15].

Primary (SS1) and secondary SS (SS2) share clinical and laboratorial similarities in different population reports, although the definition of each is clearly stated by the American European Classification Criteria [5,16]. The comparison of ocular signs and symptoms in SS1, SS2 and nonSS (NSS) was performed but not significant differences were found in the Japanese population, however, higher frequency of symptoms and extra glandular manifestations were observed in a comparison of SS1 and SS2 associated with rheumatoid arthritis in China [17,18]. The combination of ocular tests to distinguish SS1 and SS2 was not applied before. Possible differences in ocular manifestations among subtypes of the disease would be relevant to anticipate complications and customize treatment.

Considering what it is known about the mechanisms of those diseases (i.e.; inflammatory, hormone deprivation, nerve damage, metabolic deprivation, involvement of other organs and others), one can predict that they would be distinguished through the broad diagnostic tools related to DED. A similar analytical tool was used recently and recognized that biochemical pathway differences in patients with systemic lupus erythematous (SLE) compared to SS1 and systemic sclerosis (SSc) may be used as a distinctive tool among them and also explain physiopathological mechanisms in those diseases [19].

This observation is relevant because in many studies, including clinical trials, DED is evaluated as a unique homogeneous condition [20–22]. This simplification can help to explain three of the major challenging aspects of DED: 1) the discrepancy between symptoms and signs [23], 2) the difficulty to make diagnosis using one single tool or exam [24–27], and 3) lack of tools to assess the impact of a treatment for DED resulting in weak or absent conclusion in most of the systematic reviews and clinical trials [28–30].

The hypothesis of this study is that different diseases (etiologies) related to DED have distinguishable performance in different DED tests, in particular SS from other diseases and SS1 from SS2 and non SS. Therefore each condition should be monitored by the most sensitive/specific parameter(s).

Therefore, our aim is to investigate whether a set of signs and symptoms of DED is able to distinguish the common non-overlapping related systemic diseases, in particular SS and the group of tests that better identify the DED in each condition.

## Materials and methods

### Subjects

The study was approved by the Faculty of Medicine Ethics Committee (CAAE: 37688914.2.0000.5440), University of Sao Paulo and was conducted in accordance with the Declaration of Helsinki and current legislation on clinical research. Written informed consent was obtained from all subjects after explanation of the procedures and study requirements.

#### Comparison of SS and other causes of DED

This transversal study analyzed the performance of the tests currently used for diagnosis of DED among individuals belonging to five groups of diseases and a control group. The methods of enrollment, demographic and clinical data of those cases were published elsewhere, in a study that reported the sensitivity, specificity and the positive predictive value of DED tests alone and in combination for DED individuals disregarding the causes and in each of those different diseases [31]. One hundred eighty-two DED patients were recruited among consecutive patients attending the outpatient DED clinic in a referral university hospital. Individuals with the following confirmed and non-overlapping diagnosis associated with DED were included: SS diagnosed following the American-European Criteria [5], GVHD, GO, or chronic exposure to BAK preserved hypotensive drugs for glaucoma at least for one year, and DM but not diabetic retinopathy (confirmed fasting glycaemia and indirect fundoscopy).

The DED diagnostic criteria supported by different clinical studies was the following: Ocular Surface Disease Index (OSDI) score > 20 and/or Schirmer test without anesthesia (ST) <10 mm or tear break up time (TFBUT) ≤ 6 seconds and/or any of the vital staining > 3. DED diagnosis was assigned if the patient presented at least one positive test according to these parameters [32–35]. Patients were separated into five different subgroups based on their disease (i.e.; SS, GVHD, GO, DM without retinopathy, or chronic glaucoma treatment with BAK preserved eye drops), were compared throughout the study.

Twenty-four healthy individuals with similar age range and sex distribution were analyzed as the control group (CG). In this control group, subjects reporting ocular infection or allergy, ocular surgery or contact lens wear, pregnancy and lactation, or conditions with clinical overlapping of the aforementioned diseases were excluded.

#### Comparison among SS1, SS2 and non SS DED

In another transversal study, involving one hundred thirty individuals with DED, classified as SS1, SS2 and non SS by the 2002 American-European Criteria, the comparison was performed to investigate whether their performance in the DED tests were similar or capable to distinguish them by their response to a single or a combination of tests. Evaluation included the above-mentioned ocular tests and the same criteria to label them as DED was applied.

### Instrumentation and procedures

Evaluation of DED included: OSDI questionnaire [36], tear break-up time (TFBUT), corneal fluorescein staining score (CFS) and Schirmer test (ST), as described below and according to the suggested sequence [37].

#### OSDI

The OSDI is a worldly applied subjective symptom score questionnaire, recently validated Portuguese language, and used to score the frequency of DED symptoms. A Portuguese language validated version was used [36,38].

#### Tear film break-up time

Tear film break-up time (TFBUT) was measured after the instillation of 5 μl of a 2% sodium fluorescein solution over the ocular surface and spreading for 30 seconds (Allergan, Guarulhos, SP, Brazil) The value for each patient was obtained form the average of three consecutive breakup times in seconds.

#### Corneal fluorescein staining

Corneal fluorescein staining was graded in the sequence of TFBUT, observing the 2% sodium fluorescein solution impregnated in the cornea using cobalt blue illumination and following the 15-point NEI⁄ Industry scale, which consider grades of 0–3 on five regions of the cornea.

#### Schirmer test

Tear flow was measured with filter paper Schirmer test strip for 5-minutes without anesthetic in both eyes (Ophthalmos Ltd., São Paulo, SP, Brazil).

Two of the three investigators (i.e.; MA, JF and EMR) performed all measurements. The more abnormal result for each test, in the two eyes was used in the discriminant analysis.

### Statistical analysis

Descriptive statistics for data were reported as mean ± Standard Deviation (SD). Differences were considered significant at p<0.05. A multivariate data analysis was performed to classify the subjects on the pre-existing different groups based on ocular variables collected using Quadratic discriminant analysis (QDA). The QDA classifier produced a group of five discriminative functions and each subject was classified according to the cut-off point. The assumptions required to apply QDA were tested as described by Hair [39]. In order to detect potential problems with multicollinearity, the pooled within-group correlation was tested between all variables. As recommended, all the correlation coefficient were lower than 0.8 [39].

Additionally, a receiver operating characteristic curve (ROC) was built to determine the area under the curve (AUC). The AUC was used to evaluate the classification performance of individuals in different diseases [40]. All analyses were performed using JMP Pro 10.0 (SAS Institute, Cary, North Carolina).

## Results

### Comparison of SS and other causes of DED

Dry eye tests from two hundred and six individuals were analyzed. One hundred eighty-two DED subjects, with a male ratio of 18.1%, and mean age of 51.8±14.2 years, were included in this study. Based on the criteria for DED stated above, the following subgroups were formed: SS (n = 98), GVHD (n = 28), GO (n = 28), DM (n = 11), BAK (n = 17). Twenty-four healthy and non-dry eye volunteers composed the CG, with a male ratio of 29.2%, and mean age of 45.7±12.7 years.

The mean values of ocular evaluation for DED in SS individuals, in the other groups, and CG varied inside and among the different groups (Table 1).

**Table 1:** Descriptive statistics for parameters of DED in the study groups CG, BAK, DM, GO, GVHD and SS. Data expressed as mean ± SD (minimum–maximum).

Group	N	OSDI (0–100)	CFS (0–15)	TFBUT (sec)	ST (mm)
CG	24	12.8 ± 10.4 (0–45) ^b,d,e,f^	0.1 ± 0.3 (0–1) ^e,f^	10.5 ± 2.6 (5–15) ^b,c,d,e,f^	24.4 ± 9.4 (12–40) ^b,e,f^
BAK	17	34.6 ± 21.4 (2–87) ^a^	0.4 ±0.8 (0–3) ^e,f^	5.5 ± 3.4 (1–12) ^a^	13.1 ± 12.5 (2–40) ^a^
DM	11	30.0 ± 22.2 (10–73) ^a^	0.7 ±1.3 (0–3) ^e^	6.2 ± 4.4 (0–12) ^a^	11.8 ± 9.5 (3–36)
GO	28	47.1 ± 18.8 (12–79) ^a^	0.7 ±1.2 (0–4) ^e,f^	5.4 ± 3.2 (2–12) ^a^	15.7 ± 12.3 (2–40)
GVHD	28	48.2 ± 21.7 (7–85) ^a^	4.4 ±3.8 (0–15) ^a,b,c,d^	4.0 ± 2.8 (0–11) ^a^	8.4 ± 8.7 (0–35) ^a^
SS	98	48.8 ± 21.6 (0–91) ^a^	2.1 ±2.0 (0–8) ^a,b,c,d^	3.7 ± 2.4 (0–10) ^a^	9.7 ± 10.2 (0–42) ^a^

CG = Control Group; BAK = Glaucoma under Chronic Topical Treatment with Benzalkonium Chloride; DM = Diabetes Mellitus; GO = Graves’ Orbitopathy; GVHD = Graft versus Host Disease; SS = Sjögren’s syndrome; OSDI = Ocular Surface Disease Index; CFS = Corneal Fluorescein Staining; LGCS = Lissamine Green Conjunctival Staining; TFBUT = Tear Film Break-Up Time; ST = Schirmer Test; TFOsm = Tear film Osmolarity.

Kruskal–Wallis test with Dunn's multiple comparison post-hoc test.

Superscripted lowercase letters indicate statistical difference (p<0.05) vs CG^a^, BAK^b^, DM^c^, GO^d^, GVHD^e^, SS^f^.

All groups have number of subjects greater than number of variables, and only two groups have number of subjects smaller than 20 (DM and BAK). Pooled within-group correlation coefficients for all pairs of analysis revealed that the higher correlations were between ST vs TFBUT (0.47) and CFS vs TFBUT (-0.39)(Table 2).

**Table 2:** Multivariate correlation coefficients for all pairs of analysis related to the six groups compared.

	OSDI	CFS	TFBUT	ST
**OSDI**	1.00	0.29	-0.36	-0.28
**CFS**	0.29	1.00	-0.39	-0.27
**TFBUT**	-0.36	-0.39	1.00	0.47
**ST**	-0.28	-0.27	0.47	1.00

The QDA produced four discriminant functions (DF) to classify each subject into six groups. Using unstandardized canonical coefficients the discriminant functions were constructed. The eigenvalue is a ratio of the between-groups sum of squares to the within-groups or error sum of squares. It is related to effectiveness of discriminant function. Larger eigenvalues are better and they are sorted in descending order of importance (Table 3). The two first functions can explain 96.9% of the variance, and the other three remaining 3.1%. Canonical correlation provides correlation coefficient between discriminant scores on the function and groups and it was used to compare the importance of each discriminant function. The Wilks’ Lambda Test showed that the discriminant functions significantly explained the membership of the group, except the last two.

**Table 3:** Eigenvalues of each discriminant function in descending order, from DF_1_ to DF_4_. The eigenvalue is a ratio of the between-groups sum of squares to the within-groups or error sum of squares, and it's a relative measure of how different groups are on the discriminant function.

Discriminant Function	Eigenvalue	Percentage of Variance Explained	Cumulative Percentage	Canonical Correlation	Wilks’ Lambda test (p-value)
DF_1_	0.94	77.1	77.1	.70	< .0001
DF_2_	.24	19.9	96.9	.44	< .0001
DF_3_	.03	2.9	99.9	.19	.30
DF_4_	.001	.1	100.0	.03	.91

The standardized canonical discriminant function coefficient shows the contribution of each variable to the discriminant function to compare variables in different scales (Table 4). Large values on standardized coefficient reflect greater discriminant ability to their corresponding variables. Considering the whole groups and tests included in the quadrant discriminant analysis (QDA), the most valuable test for classification was TFBUT, followed by CFS, OSDI, and ST.

**Table 4:** Standardized canonical discriminant function coefficient comparing variables in different scales (variables were adjusted by subtraction of its mean value and division by its standard deviation).

Signs and Symptoms	DF_1_	DF_2_	DF_3_	DF_4_
TFBUT	0.62	0.45	0.18	0.73
OSDI	-0.48	-0.32	0.68	0.48
CFS	-0.25	0.99	0.16	-0.10
ST	0.21	-0.01	0.74	-0.72

OSDI = Ocular Surface Disease Index; CFS = Corneal Fluorescein Staining; TFBUT = Tear Film Break-Up Time; ST = Schirmer Test

Those variables combined were able to fit 49% of individuals in their actual group (Table 5).

**Table 5:** Distribution and discrimination of diseases related to DED. The bold numbers on the rows present the individuals classified in their original groups and the numbers in the cells aside at the same row reveal their misclassification based on the six tests for DED applied to those individuals.

Actual Group	Predicted Group						Total	% Correct
	CG	BAK	DM	GO	GVHD	SS		
CG	22	1	1	0	0	0	24	91.7
BAK	4	8	2	2	0	1	17	72.7
DM	2	3	4	0	0	2	11	36.4
GO	2	9	3	10	1	3	28	35.7
GVHD	0	1	2	3	11	11	28	39.3
SS	4	20	1	16	11	46	98	46.9

CG = Control Group; BAK = Glaucoma under Chronic Topical Treatment with Benzalkonium Chloride; DM = Diabetes Mellitus; GO = Graves’ Orbitopathy; GVHD = Graft versus Host Disease; SS = Sjögren’s syndrome.

Only two groups have most of their individuals discriminated to their actual group. The CG had 91.7% of correct discrimination, followed by BAK, 72.7%, SS 46.9%, DM 36.4%, GVHD 39.3% and GO 35.7% (Table 5).

**Fig 1:**
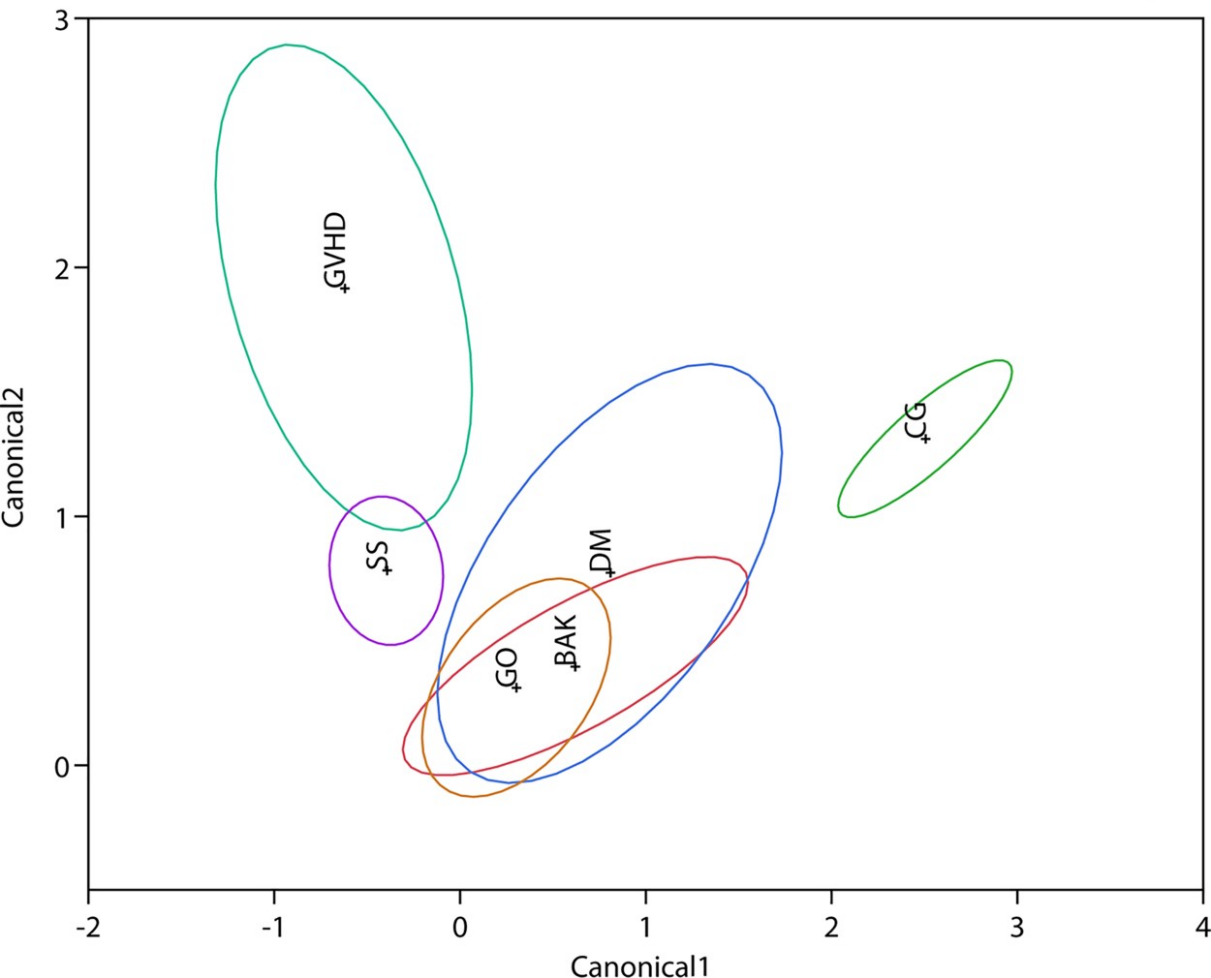
Discrimination map of the individuals of the different groups of diseases related to DED and their similarities regarding the six tests used to evaluate DED. The center of each ellipse is marked by the abbreviation of each group studied (CG = Control Group; BAK = Glaucoma under Chronic Topical Treatment with Benzalkonium Chloride; DM = Diabetes Mellitus; GO = Graves’ Orbitopathy; GVHD = Graft versus Host Disease; SS = Sjögren’s syndrome) and their limits indicate the confidence interval of that group. X and Y axis numbers (Canonical 1 and 2, respectively) indicate the geometric distance of the discriminant analysis among the groups.

The most overlapping conditions were GVHD and SS, where 39.3% of patients with GVHD not discriminated from SS patients. An important part of SS group (20.4%) was misclassified as BAK. Also, GO and BAK were overlapped and both had cases not properly discriminated from DM, using the four tests to diagnosis DED (Table 5 and Fig 1).

**Fig 2:**
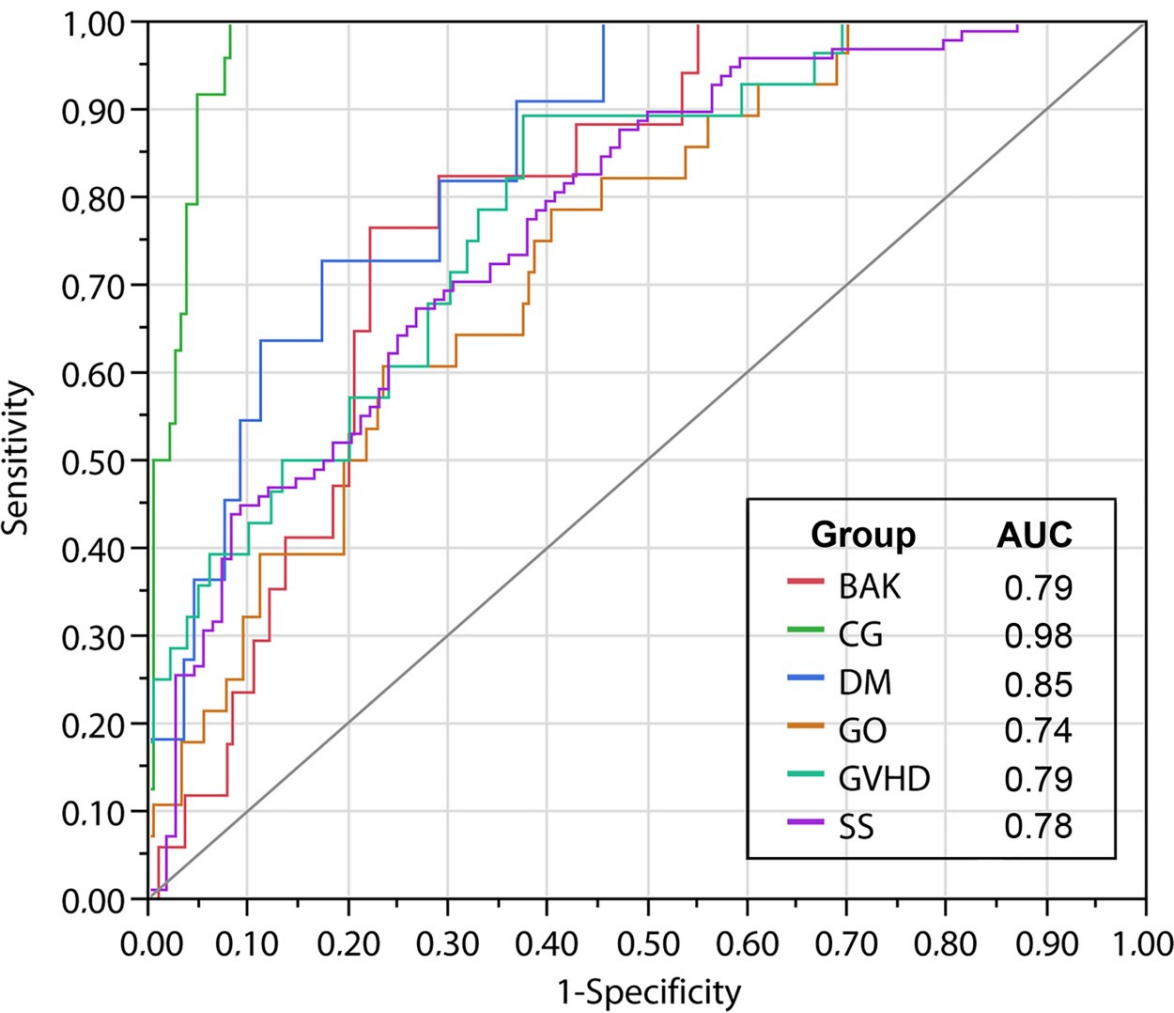
ROC curve (Receiver Operating Characteristics) revealing the frequency of discrimination among those six groups (CG = Control Group; BAK = Glaucoma under Chronic Topical Treatment with Benzalkonium Chloride; DM = Diabetes Mellitus; GO = Graves’ Orbitopathy; GVHD = Graft versus Host Disease; SS = Sjögren’s syndrome), using six tests to investigate DED. The area under the curve (AUC) shows the percentage of correct discrimination associating sensitivity (Y) and 1-specificity (X).

Following the criteria of the ROC curve, applying those six DED tests to discriminate the six diseases associated with DED, again the group with better discrimination was the CG with 97.6%, followed by DM with 84.9%, GVHD with 79.4%, BAK with 78.7, SS with 77.5% and GO with 73.9%. The groups with higher frequency of overlapping of individuals were DM, GO and BAK. Dismissing those three groups the percentage of correct fitting the individuals in their actual groups was 65% (Fig 2).

### Comparison among SS1, SS2 and non SS DED

One hundred thirty individuals were analyzed to investigate whether DED tests can distinguish individuals from SS1, SS2 and NSS groups. The female ratio was 93.5%, and mean age 53.7 ± 14.9 years. Based on the criteria for DED and American-European Consensus for SS, the following groups were composed by 57 individuals in the SS1), 41 individuals (SS2), 32 individuals (NSS).

The mean values of the DED tests in the three groups were significantly different, except for OSDI that was lower in the NSS group but presented large variability in all the groups (p>0.05). NSS differ from SS1 in CFS score, from SS2 in TFBUT mean values and from both SS1 and SS2 in ST (Table 6).

**Table 6:** Descriptive statistics for parameters of DED in the study subgroups SS1, SS2 and Non-SS (NSS). Data expressed as mean ± SD (minimum–maximum).

Group	N	OSDI (0–100)	CFS (0–15)	TFBUT (sec)	ST (mm)
NSS	32	40.1 ± 19.6 (12–84)	1.0 ± 1.8 (0–6) ^b^	5.2 ± 3.1 (0–11) ^c^	17.3 ± 10.8 (2–35) ^b,c^
SS1	57	48.0 ± 22.5 (0–91)	2.4 ± 2.2 (0–8) ^a^	3.9 ± 2.3 (0–10)	9.1 ± 9.9 (0–35) ^a^
SS2	41	49.9 ± 20.4 (15–91)	1.7 ± 1.6 (0–5)	3.5 ± 2.5 (0–10) ^a^	10.5 ± 10.8 (0–42) ^a^

SS1 = Primary Sjögren’s syndrome; SS2 = Secundary Sjögren’s syndrome; Non-SS = Non Sjögren’s syndrome

OSDI = Ocular Surface Disease Index; CFS = Corneal Fluorescein Staining; LGCS = Lissamine Green Conjunctival Staining; TFBUT = Tear Film Break-Up Time; ST = Schirmer Test; TFOsm = Tear film Osmolarity

Kruskal–Wallis test with Dunn's multiple comparison post-hoc test.

Superscripted lowercase letters indicate statistical difference (p<0.05) vs nonSS^a^, SS1^b^, SS2^c^.

The QDA produced two discriminant functions to classify each subject into three groups. The first discriminate function (DF_1_) explained 79.4% of variance.

**Fig 3:**
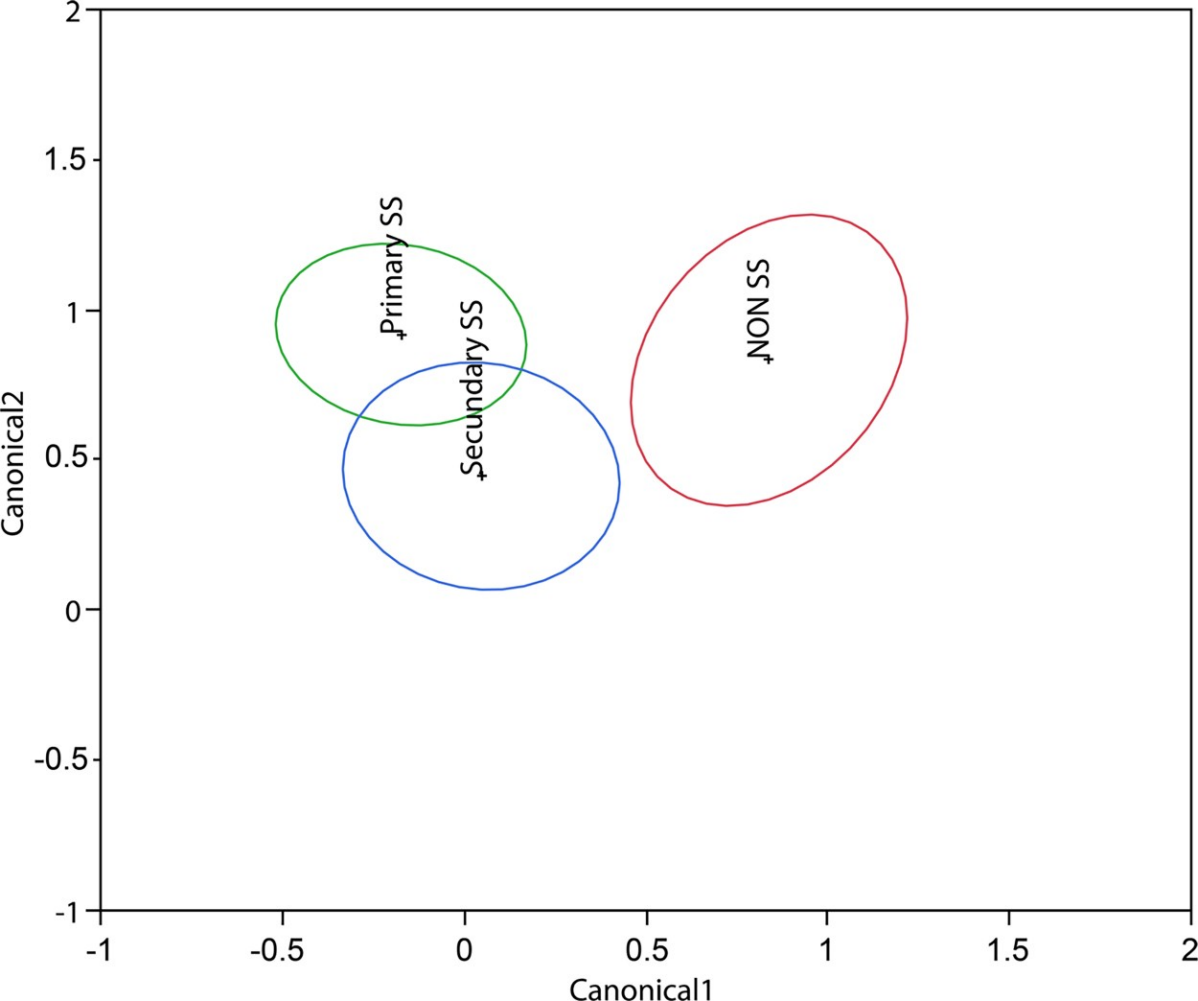
Discrimination map of the individuals with DED and Sjögren’s syndrome (SS). Groups Primary SS (SS1), Secondary SS (SS2) and NSS and their similarities regarding the four tests used to evaluate DED. The center of each ellipse is marked by the abbreviation of each group studied (SS1 Group; SS2 group and NSS). The limits indicate the confidence interval of that group. X and Y axis numbers (Canonical 1 and 2, respectively) indicate the geometric distance of the discriminant analysis among the groups.

The quadratic discriminant analysis revealed that the combined tests correctly classified 54.6% of the patients in their groups (Wilks' Lambda = 0.82; p = 0.0015). The area under the ROC curve better classified NSS (79.5%), followed by SS2 (74.4%) and SS1 (69.4%). The most relevant variables were respectively ST, CFS, TFBUT and OSDI (Fig 3).

**Fig 4:**
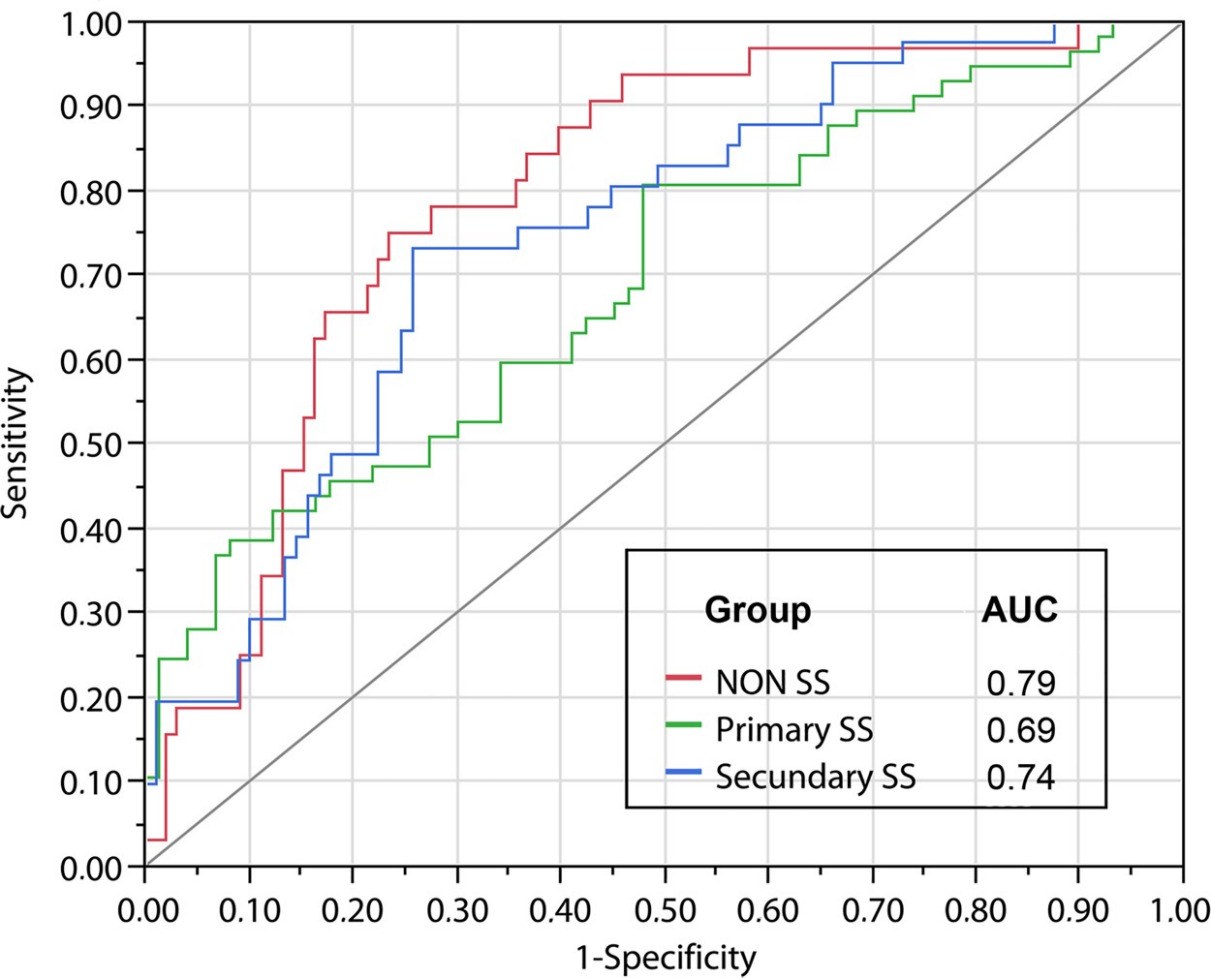
ROC curve (Receiver Operating Characteristics) revealing the frequency of discrimination among the three groups regarding Sjögren’s syndrome (SS) and dry eye tests (SS1, SS2 and NSS), using four tests to investigate DED. The area under the curve (AUC) shows the percentage of correct discrimination associating sensitivity (Y) and 1-specificity (X).

The ROC curve, applying the four DED tests to discriminate the three diseases associated with DED, again the group with better discrimination was the SS2 with 74.4%, followed by NSS with 79.5%, and SS1 with 69.4%. Higher frequency of overlapping was found between SS1 and SS2. The NSS group was 59.4% correctly classified, while SS1 and SS2 were correctly classified in 49.1% and 58.5% of the cases, respectively. SS1 and SS2 were misclassified as NSS in 24.5% and 26.3%. (Fig 4).

## Discussion

The present work shows that common used diagnostic tests for DED are capable to discriminate different etiologies associated to this condition. Although it is not clear the exact relationship of those DED tests and the mechanisms of the disease, [37] it supports the hypothesis that different diseases cause DED toward different mechanism and it may be reflected in the performance of combined tests.

DED tests have a long history, some modern achievements, but are in fact measuring parameters related to the tear film secretion, ocular surface changes and sensation [27,37]. Those tests are known to present variability and overlapping between DED and healthy individuals, even observing groups of diseases [24,41]. The present work explored the discriminant analysis to differentiate distinct groups of DED and the controls, considering the variability of test results and difficulties to diagnose DED using a single sign or test.

Discriminant analysis is an appropriate multivariate technique that associates multiple independent variables to a categorical dependent variable, and its application has been used in health sciences and DED [41–43]. In a prior study this analysis revealed that tear osmolarity alone was able to distinguish dry eye from healthy individuals with an accuracy of 89% [41]. In the first part of the present work five discriminant functions were constructed to classify the subjects on the six groups based on ocular signs. The Wilks’ lambda revealed that the first three functions were significant and explained a total variance of 96.9%.

Higher standardized coefficient, in absolute value, reflects greater discriminant ability to their corresponding variables. Considering the tests used here, it is acceptable that OSDI was the most useful test to discriminate the diseases. DED patients are diagnosed when seeking for help for their symptoms. As shown in a previous publication, OSDI had higher mean scores in inflammatory diseases as SS, GVHD and GO, and lower in DM [24]. The cornea is the most innervated surface of the body and provides feedback for the lacrimal secretory system [44,45]. Those diseases trigger changes in the ocular surface environment in a distinct way based on their mechanisms, whether inflammatory (e.g. SS and GVHD), neural or hormonal (e.g.; DM, BAK) and are better detected by certain groups of exams.

It is understandable that SS is the best-discriminated disease. SS has a very complex system of diagnosis with six items and several exclusion criteria [5]. The diagnostic process eliminates any conditions with confounding factors. GVHD was called Sjögren’s like syndrome in the past and appeared with certain overlapping here, but it has a clear etiology and distinguished epidemiological aspects, not explored in this work [46,47].

DM, GO and BAK groups also presented a substantial overlap, probably due to the multiple factors involved in their changes regarding DED, which includes neuropathic damage, hormone impairment and environmental factors [11,48–50].

The present analysis also identified redundant variables in certain groups of diseases. CFS and LGCS showed stronger correlation coefficients. Previous authors investigated the combination of markers, but also pointing the discrepancies among them [51,52].

The higher AUC revealed in the ROC analysis agreed with the classification distribution. They showed that CG was the best identified by the tests and the last three groups with more overlapping classification were GVHD, GO, BAK. The possible explanation is the broad spectrum of the disease among the individuals recruited for this study. The time-length and therapeutic interventions may have contributed to this finding.

There are few studies that have used the discriminant analysis to address hypothesis on DED. [41, 53] On those studies, comparisons were performed between two groups, DED patients and non-DED subjects. Khanal et al.[41] reported an AUC of 0.95, indicating that the discriminant function was a reliable diagnostic tool for dry eye. Kaido M et al.[53] applied the discriminant function to distinguish DED patients from non-DED subjects using functional visual acuity and they obtained AUC of 0.735. Although the variables analyzed were different, we could compare to our results and consider it as similar (AUC = 0.98, for controls). It is important to note that in the present study the number of groups is higher (more than two), with different etiologies for DED. Thus, the AUC value should be interpreted here as the ability of the combined signs and symptoms, to distinguish a particular group from all others. Therefore, this study does not intend to use dry eye tests to identify which etiology the subject belongs to. On the contrary, we proposed a new way of visualizing the distribution of the set of signs and symptoms of dry eye related to its etiologies.

The quadratic discriminant analysis was not previously attempted in the diagnosis of SS. Considering the extensive panel of tests to define the diagnosis and the long-term implications of carrying this condition with its proper diagnosis, we considered useful to identify the ocular tests more sensitive to differentiate the SS (both SS1 and SS2) from NSS individuals. Despite all the limitations of ST, its values were significantly different between SS and NSS. Further efforts on the identification of useful tests may help to improve the diagnostic and follow up panel.

Some potential limitations of this study must be pointed out. First, the discriminant analysis was based on signs that may present variability day after day and also in the same day. It would compromise the results, however, data was randomly harvested and therefore they reproduce the real conditions of patients presented to the outpatient clinic. Indeed, there were patients under treatment or not in all the groups. Treatment for the underlying condition may also influence on the tests, but that was present in all groups. The second issue is associated with the DED inclusion criteria. The lack of a gold standard and arbitrary selection of tests and threshold may induce bias. The authors adopted the most used tests and cut-off numbers widely validated in the literature avoiding to constrain the selection of DED patients for each group [31–33,35,37,54].

The intention of this study was to investigate the performance of ocular signs set in the different known diseases; therefore no cross-validation was applied.

In conclusion, individuals from five different groups of diseases associated with DED have a moderate capacity to be fitted in their actual group based on traditional DED diagnostic tests. Together, the combined DED tests classified the individuals in their correct group from 41.2 to 69.6% of cases. These findings showed that the actual DED tests, even when combined present a limited capacity to discriminate different causes of DED. In the same way, the DED tests distinguished SS1 and SS2 from NSS individuals with DED. Taken together, our results indicate the need for further studies to better identify tests as biomarkers of the physiopathology of DED and therefore, to evaluate more specific therapies and anticipate the prognosis for DED.
